# Biomechanical Effects of Lateral Bending Position on Performing Cervical Spinal Manipulation for Cervical Disc Herniation: A Three-Dimensional Finite Element Analysis

**DOI:** 10.1155/2018/2798396

**Published:** 2018-06-11

**Authors:** Xuecheng Huang, Linqiang Ye, Zixian Wu, Lichang Liang, Qianli Wang, Weibo Yu, De Liang, Xiaobing Jiang

**Affiliations:** ^1^First School of Clinical Medicine, Guangzhou University of Chinese Medicine, Guangzhou 510405, China; ^2^Department of Spinal Surgery, First Affiliated Hospital of Guangzhou University of Chinese Medicine, Guangzhou 510405, China; ^3^Shenzhen Hospital of Guangzhou University of Chinese Medicine, Shenzhen 518034, China; ^4^Department of Orthopaedic and Traumatology, Tongde Hospital of Zhejiang Province, Hangzhou 310012, China; ^5^Laboratory Affiliated to National Key Discipline of Orthopaedic and Traumatology of Chinese Medicine, Guangzhou University of Chinese Medicine, Guangzhou 510405, China

## Abstract

**Background:**

Most studies report that the common position of cervical spinal manipulation (CSM) for treating symptomatic cervical disc herniation (CDH) is lateral bending to the herniated side. However, the rationality of lateral bending position on performing CSM for CDH is still unclear.

**Objective:**

The purpose of this study is to investigate the biomechanical effects of lateral bending position on performing CSM for CDH.

**Methods:**

A finite element (FE) model of CDH (herniated on the left side) was generated in C5-6 segment based on the normal FE model. The FE model performed CSM in left lateral bending position, neutral position, and right lateral bending position, respectively. Cervical disc displacement, annulus fiber stress, and facet joint stress were observed during the simulation of CSM.

**Results:**

The cervical disc displacement on herniated side moved forward during CSM, and the maximum forward displacements were 0.23, 0.36, and 0.45 mm in left lateral bending position, neutral position, and right lateral bending position, respectively. As the same trend of cervical disc displacement, the annulus fiber stresses on herniated side from small to large were 7.40, 16.39, and 22.75 MPa in left lateral bending position, neutral position, and right lateral bending position, respectively. However, the maximum facet stresses at left superior cartilage of C6 in left lateral bending position, neutral position, and right lateral bending position were 6.88, 3.60, and 0.12 MPa, respectively.

**Conclusion:**

Compared with neutral position and right lateral bending position, though the forward displacement of cervical disc on herniated side was smaller in left lateral bending position, the annulus fiber stress on herniated side was declined by sharing load on the left facet joint. The results suggested that lateral bending to the herniated side on performing CSM tends to protect the cervical disc on herniated side. Future clinical studies are needed to verify that.

## 1. Introduction

Cervical disc herniation (CDH) is a common cause of cervical radiculopathy which occurs in approximately 85.4 of every 100000 persons [1, 2]. Symptoms of CDH which include pain and disability innervated by the nerve root can arise from the nerve root compression, inflammation, or both [2]. Patients with symptomatic CDH are initially treated conservatively, with surgery reserved for those cases that remain unresponsive to conservative treatment. Up to 90% of patients with symptomatic CDH will have significant improvement in symptoms with conservative treatment [2].

Cervical spinal manipulation (CSM) is one of the important conservative treatments for symptomatic CDH [3–5]. Studies reported that CSM reduces pain in patients with symptomatic CDH [6–10]. The manipulation commonly included two key procedures of rotation to the healthy side and lateral bending to the affected (herniated) side [6–10]. Wu et al. suggested that compression of the nerve root is relieved by a small displacement between it and the herniated disk during CSM with rotation to the healthy side [11, 12]. However, the rationality of lateral bending to the herniated side on performing CSM for CDH is still unclear [13].

The purpose of the present study is to compare the biochemical effects of different lateral bending positions on performing CSM through three-dimensional finite element analysis, so as to evaluate the rationality of lateral bending to the herniated side on performing CSM for CDH.

## 2. Materials and Methods

### 2.1. A Finite Element Model of Intact Cervical Spine (C3–7)

Following the methods of Zhong Jun Mo et al. 2014 [14], a normal three-dimensional finite element (FE) model of intact cervical spine was built using digitized image data of a C3-7 motion segment. The image data of C3-7 was obtained by a 64-detector CT scanner (Discovery CT750 HD, GE Healthcare, USA) at 0.625-mm interval from a healthy female volunteer (25 years old, 55 kg, and 165 cm) without any radiographic evidence of degenerative sign.

The slice images were imported into medical image processing software (Mimics 10.1, Materialise Inc., Belgium) to reconstruct the vertebrae geometry volume of C3–7. The geometry of other structures (the annulus fibrosis, nucleus pulpous, facet cartilage), which were difficult to separate from the CT images, was modeled using the solid modeling software, SolidWorks 2014 (SolidWorks Corp, Dassault Systèmes, Concord, M A).

**Figure 1:**
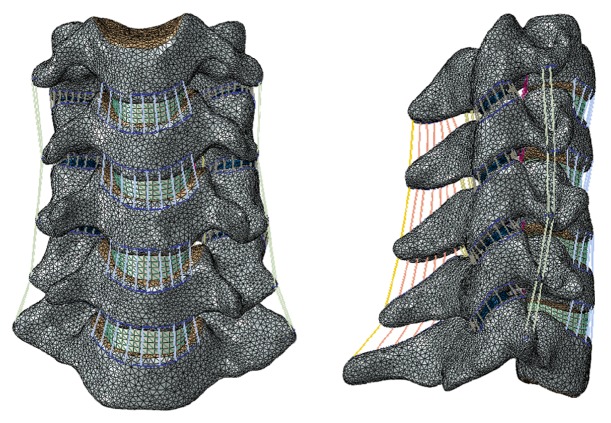
The normal FE model of C3-7.

Finite element modeling software (ABAQUS 2016, Simulia Inc., USA) was used to build and mesh the cervical spine components. The vertebrae were made up of a solid volume (cancellous bone) and a layer of shell (cortical bone and endplate) with a thickness of 0.4 mm [14, 15]. The intervertebral disc was constructed as a continuum structure partitioned into nucleus pulposus and annulus fibrosus. The nucleus pulposus was 43% of the total disc volume and located slightly posterior to the center of the disc [16]. The annulus fibrosus was modeled as a composite structure: the annulus ground substance reinforced by inclined fibers acting at approximately ±30° from the transverse plane [17]. Seven intervertebral ligaments were incorporated, including anterior longitudinal ligament (ALL), posterior longitudinal ligament (PLL), capsular ligament (CL), flaval ligament (FL), interspinous ligament (ISL), supraspinous ligament (SSL), and transverse ligament (TL) with the suggested insertion site [18]. The cancellous bone was meshed into tetrahedron elements (C3D4), while the cortical element was meshed into triangle shell elements (S3) [14]. The annulus ground substance, the nucleus pulposus, and anterior plate were meshed into hexahedron elements (C3D8R) [14]. All the ligaments were modeled as tension-only axial connector, and the annulus fiber was meshed as tension-only truss elements (T3D2) [14]. In the FE model of intact cervical spine, the total number of nodes and elements was 216287 and 966930, respectively (Figure 1). Convergence within 1% was achieved in the intact model to ensure that the results were not relevant to the mesh density [14, 15].

### 2.2. Validation of the Normal FE Model of C3-7 and C5-6

The assigned material properties (Table 1) selected from various sources in the literature were assumed to be linear, homogeneous, and isotropic [19–21]. Tied contact interfaces were used to ensure that the disc and ligament were attached to the vertebra, preventing any relative movement during the simulations. Surface-based, finite-sliding contact with a friction coefficient 0 was defined for facet joints [22]. The validation of the normal model was conducted according to the published FE model and human cadaveric cervical spines. For model validation of C3-7, the inferior endplate of C7 was fixed at six degrees of freedom in the same way as in vitro experiments [23, 24]. A follower preload of 50N was applied to the superior endplate center of C3 to simulate the head weight in the normal FE model of C3-7 [15]. In addition to the follower preload, a moment of 1.0Nm applied to the superior endplate center of C3 to simulate flexion, extension, lateral bending, and axial rotation motions in this model [24]. Furthermore, the FE model of C5-6 was also validated for using the same experimental and simulated loading protocols, except the follower preload of 73.6N and the moment of 1.8Nm [23].

### 2.3. Simulation of Cervical Disc Herniation in C5–6 Spinal Motion Segment

**Figure 2:**
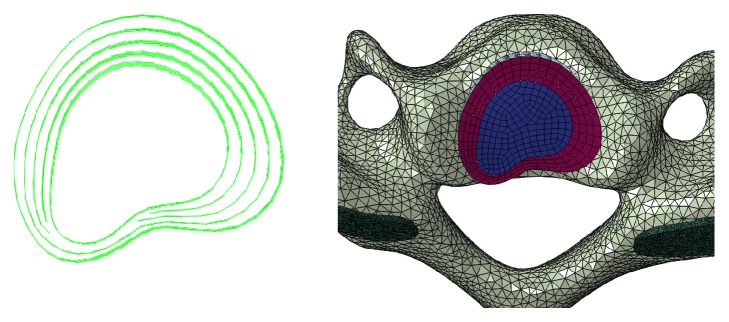
The cervical disc herniation model in C5-6.

Similar to simulation methods reported by Hussain et al. [25], the FE model of the degenerative cervical spine was generated based on the developed FE model of the normal cervical spine. The cervical disc herniation (herniated on the left side) was simulated in C5-6 spinal motion segment. The changes in geometry and material properties (Table 2) used to simulate the degeneration were adapted from the clinical classification of degeneration of the cervical spine and the results of the previously published literature [18, 25, 26]. For the herniated disc of C5-6, the left posterolateral annulus was weakened as mid-then-outer annulus fibers tear [27], allowing herniation of nuclear material into the outer annular structure as a contained protrusion, and part of the disc pass into the vertebral canal space as an extrusion, as shown in Figure 2.

The Simulation and Loading of CSM in C5–6 with CDH

In this research, we used the material properties of moderately degenerated disc to investigate the biomechanical effects of lateral bending position on performing CSM for CDH. The mechanical loading steps in sequence to simulate CSM were as follows [12]:

1. The inferior endplate of C6 was fixed.
2. The C5 vertebra, along with the entire model, was rotated 2°, 0, -2° around y-axis to simulate the left lateral bending, neutral, and right lateral bending positions, respectively.
3. In the left lateral bending, neutral, and right lateral bending positions, the cervical FE model was rotated 2° to the right side (the opposite side of CDH) around z-axis (vertical axis) to simulate rotation to the right side, respectively.
4. The cervical FE model continued to rotate a further 0.5° to the right side within 0.15 seconds so as to simulate the high-velocity, low-amplitude CSM.

### 2.4. Analysis

Cervical disc displacement, annulus fiber stress (von Mises stress) and facet joint stress (von Mises stress) were observed during the simulation of CSM.

## 3. Results

### 3.1. Validation of the Normal FE Model

**Figure 3:**
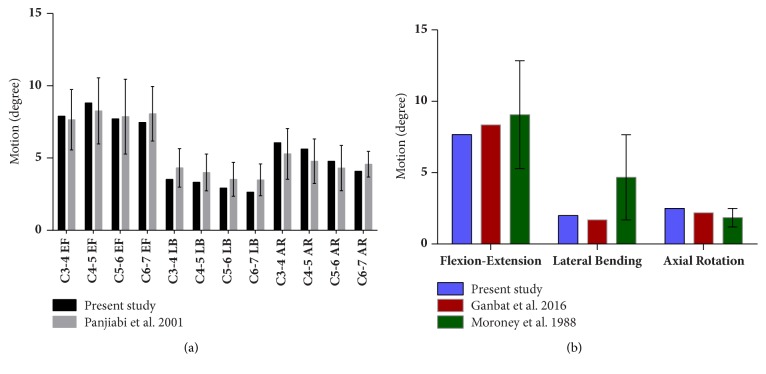
Validation of the normal FE model of C3-7 and C5-6.

The range of motion (ROM) of each functional spinal unit in the FE model of C3-7 is shown in Figure 3(a), which is consistent with in vitro experimental results [24]. Under 1.0 Nm moment and 50N follower load, the overall ROM of C3-7 was 17.53° in flexion, 10.01° in extension, 8.96° in axial rotation, and 11.05° in lateral bending, respectively. The results of C5-6 FE model validation shown in Figure 3(b) were also similar to the previous FE model (Ganbat et al.) [19] and in vitro experimental data (Moroney et al.) [23].

### 3.2. Cervical Disc Displacement

**Figure 4:**
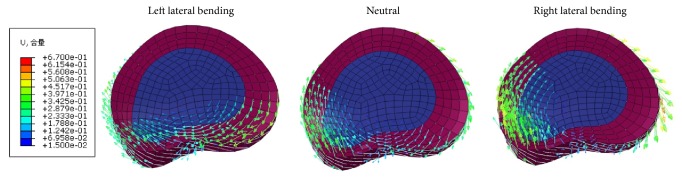
Cervical disc displacement in the left lateral bending, neutral, and right lateral bending positions during CSM.

When performing CSM, the left posterolateral (the herniated side) cervical disc moved left, forward, and up in three positions: left lateral bending, neutral, and right lateral bending (Figure 4). The maximum forward displacements of left posterolateral (the herniated side) cervical disc were 0.23, 0.36, and 0.45 mm in the left lateral bending, neutral, and right lateral bending positions, respectively.

### 3.3. Annulus Fiber Stress

**Figure 5:**
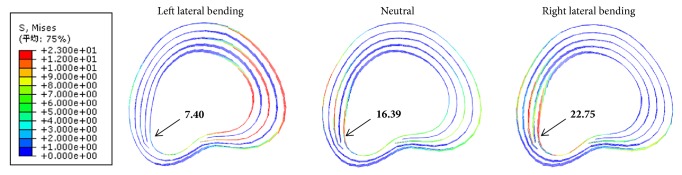
Annulus fiber stresses in the left lateral bending, neutral, and right lateral bending positions during CSM.

When performing CSM, the maximum annulus fiber stresses were 22.52, 17.61, and 23.60 MPa in left lateral bending position, neutral position, and right lateral bending position, respectively. The distribution of annulus fibers stress in left lateral bending position was concentrated on the right posterolateral (the heathy side) of cervical disc. However, the distributions of annulus fibers stress in neutral position and right lateral bending position were both concentrated on the left posterolateral (the herniated side) of cervical disc (Figure 5). And the annulus fiber stress of the same node on herniated side was 7.40 MPa in left lateral bending position, which was smaller than those in neutral position (16.39 MPa) and in right lateral bending position (22.75 MPa) while performing CSM (Figure 5, black arrows).

### 3.4. Facet Joint Stress

**Figure 6:**
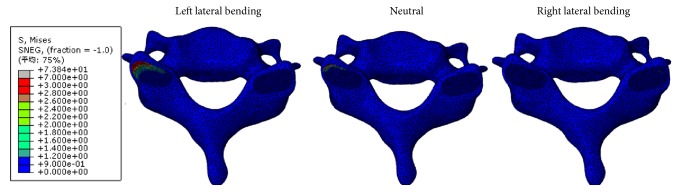
Facet joint stresses in the left lateral bending, neutral, and right lateral positions bending during CSM.

When performing CSM, the maximum facet stresses at left superior cartilage of C6 in left lateral bending position, neutral position, and right lateral bending position were 6.88, 3.60, and 0.12 MPa, respectively (Figure 6).

## 4. Discussion

Cervical disc herniation is most common at the C5-6 level [28, 29]. Patients with symptomatic CDH have good outcome associated with performing CSM at the level of CDH with a proper procedure [30]. However, some reports showed that the aggravating cervical disc rupture can occur during a process of CSM with preexisting CDH under the wrong manipulative technique [31–33]. Rotation to the healthy side and lateral bending to the herniated side are the common procedures on performing CSM for CDH. However, the rationality of lateral bending position is still unclear. In the present study, we first established a three-dimensional FE model of CDH (herniated on the left side) in C5-6 segment and then simulated CSM with rotation to the right side (healthy side) in left lateral bending position, neutral position, and right lateral bending position, to evaluate the biomechanical effects of lateral bending to the herniated side during CSM.

Interestingly, we discovered that the cervical disc on herniated side moved forward during CSM in three positions: left lateral bending, neutral, and right lateral bending. The maximum forward displacements were 0.23, 0.36, and 0.45 mm in left lateral bending position, neutral position, and right lateral bending position, respectively. It reconfirmed that a small forward displacement was generated on the left side (herniated side) of cervical disk during CSM with rotation to the right side (healthy side) [12]. And it suggested that the release of the compressed nerve root from small to large was left lateral bending position, neutral position, and right lateral bending position, respectively.

As the same trend of cervical disc displacement, the annulus fiber stress on herniated side from small to large was 7.40, 16.39, and 22.75 MPa in left lateral bending position, neutral position, and right lateral bending position, respectively. The distribution of annulus fibers stress in left lateral bending position was concentrated on the right posterolateral (the healthy side) of cervical disc. However, the distributions of annulus fibers stress in neutral position and right lateral bending position were both concentrated on the left posterolateral (the herniated side) of cervical disc. It suggested that the cervical disc on herniated side may be damaged with high annulus fibers stress during CSM [34]. Compared with left lateral bending position, the left posterolateral (the herniated side) of cervical disc is acted upon by larger annulus fibers stresses in neutral position and right lateral bending position, which could lead to more torsion on the herniated side and, thereby, may cause injury to the annulus fibrosus and therefore aggravation of disc herniation [35–37].

The facet joint stress showed the opposite trend with the cervical disc displacement and annulus fiber stress. The maximum facet stresses at left superior cartilage of C6 were 6.88, 3.60, and 0.12 MPa in left lateral bending position, neutral position, and right lateral bending position during CSM. As we known, facet joints play a role in mechanical function which can contribute to spinal stability and load sharing between spinal columns [38, 39]. The left facet joint contact force increased in left lateral bending position, rotation to right side, or combination of both [40]. In the present study, facet stress at left superior cartilage of C6 was largest in left bending position during CSM with rotation to right side. In other words, the left facet joint sustained more mechanical loading and restricted segmental motion in left bending position with rotation to right side.

Compared with neutral position and right lateral bending position, though the forward displacement of cervical disc on herniated side was smaller in left lateral bending position, the annulus fiber stress on herniated side was declined by sharing load on the left facet joint. In summary, lateral bending to the herniated side on performing CSM for treating CDH tends to protect the cervical disc on herniated side during such manipulation. Future clinical studies are needed to verify the biomechanical effects of lateral bending position on performing CSM for treating CDH.

## 5. Limitations

The present study has certain shortcomings. First, the current results were based on the FE model of a healthy female who did not have cervical disc herniation. The simulation of cervical disc herniation in C5-6 segment was made by the model adjustment, but other parameters and geometry did not change from the normal FE model. The interpretation of the results should be cautious because the results were drawn from the adjusted model. Second, the loading conditions were highly idealized and could not represent the complicated condition of cervical spinal manipulation. Therefore, it should be kept in mind that the present results were driven by these assumptions. However, the results of FE analysis represent trends rather than precise values because of the necessary simplifications and assumptions concerning the geometry, material properties of the different tissues, contact behavior, and applied loads [41]. Third, the musculature's effect on the stability of the cervical spinal was not considered in the present study. It remains to be determined how much the current findings would vary if this limitation is appropriately addressed in our future FE models.

## 6. Conclusions

Compared with neutral position and right lateral bending position, though the forward displacement of cervical disc on herniated side was smaller in left lateral bending position, the annulus fiber stress on herniated side was declined by sharing load on the left facet joint. In summary, lateral bending to the herniated side on performing CSM for treating CDH tends to protect the cervical disc on herniated side. Future clinical studies are needed to verify that.

## Figures and Tables

**Table 1:** Material properties of the C3-7 finite element model.

Component	Young's modulus (MPa)	Poisson's ratio	Cross-sectional area (mm^2^)
Cortical bone	12000	0.29	-
Cancellous bone	100	0.29	-
Endplate	1200	0.29	-
Annulus ground substance	3.4	0.45	-
Annulus fiber	450	0.40	-
Nucleus pulposus	1	0.49	-
Cartilage	10.4	0.40	-
ALL	10	0.30	12
PLL	10	0.30	45
CL	10	0.30	14
FL	1.5	0.30	5
ISL	1.5	0.30	13
SSL	1.5	0.30	13
TL	17	0.30	10

ALL: anterior Longitudinal ligament; PLL: posterior longitudinal ligament; CL: capsular ligament; FL: flaval ligament; ISL: interspinous ligament; SSL: supraspinous ligament; TL: transverse ligament.

**Table 2:** Elastic tissue material properties of the AF and NP.

Description	Element type	Young's modulus (MPa)	Poisson's ratio
Normal disc			
AF	3D solid (4 node)	2.50	0.40
NP	3D solid (4 node)	1.50	0.49
Moderately degenerated disc			
AF	3D solid (4 node)	2.50	0.40
NP	3D solid (4 node)	2.00	0.49
Severely degenerated disc			
AF	3D solid (4 node)	5.00	0.20
NP	3D solid (4 node)	4.00	0.25

AF Young modulus represents the modulus of lateral AF.

AF: annulus fibrosus; NP: nucleus pulposus.

## Data Availability

The data used to support the findings of this study are available from the corresponding author upon request.
